# A prospective randomized double-blind study comparing the dose-response curves of epidural ropivacaine for labor analgesia initiation between parturients with and without obesity

**DOI:** 10.3389/fphar.2024.1348700

**Published:** 2024-02-16

**Authors:** Xiao-Dong Huang, Xiao-Xiao Qiu, He-Jie Wang, Xia-Fang Jin, Fei Xiao

**Affiliations:** ^1^ Department of Anesthesiology, Hangzhou Women’s Hospital, Hangzhou Maternity and Child Healthcare Hospital, Hangzhou First People’s Hospital Qianjiang New City Campus, Zhejiang Chinese Medical University, Hangzhou, China; ^2^ Department of Anesthesiology, Wenzhou Hospital of Integrated Traditional Chinese and Western Medicine, Wenzhou, China; ^3^ Department of Anesthesiology, Jiaxing University Affiliated Women and Child Hospital, Jiaxing, China

**Keywords:** ropivacaine, obesity, dose-response, labor analgesia, epidural

## Abstract

**Background:** Previous studies have explored the median effective concentration (EC50) of ropivacaine for labor epidural analgesia in parturients with obesity. However, the clinical relevance of the 90% effective concentration (EC90) remains unclear. This study aimed to determine and compare the dose–response curve of epidural ropivacaine for labor analgesia between parturients with and without obesity.

**Methods:** Parturients were divided into two groups based on body mass index (BMI): group N, consisting of parturients with BMI <30 kg/m^2^, and group O, consisting of parturients with BMI >30 kg/m^2^. Within each group, the patients were randomized to receive one of five concentrations (0.0375%, 0.075%, 0.1125%, 0.15%, or 0.1875%) of epidural ropivacaine for labor analgesia. Analgesia was induced with a loading dose of 15 mL of the assigned concentration. Visual analogue scale (VAS) scores were recorded at baseline and 30 min post-dose to calculate the response (%) using the formula [(baseline VAS pain score—VAS pain score at 30 min)/baseline VAS pain score] ×100%. The EC50 and EC90 values were determined via nonlinear regression analysis.

**Results:** The EC50 and EC90 values of ropivacaine were 0.061% (95% confidence interval [CI], 0.056%–0.066%) and 0.177% (95% CI, 0.152%–0.206%) in group N and 0.056% (95% CI, 0.051%–0.061%) and 0.161% (95% CI, 0.138%–0.187%) in group O, respectively. No significant differences were observed in the EC50 and EC90 values between the two groups (*p*-values = 0.121 and 0.351, respectively.

**Conclusion:** In conclusion, within the parameters of this study, our findings suggest that obesity, characterized by a mean BMI value of 30.9, does not significantly influence the EC50 and EC90 values of epidural ropivacaine for labor analgesia. Further investigations are warranted to elucidate the dose-response relationship between ropivacaine and obesity with higher BMI values.

**Clinical trial registration:** https://www.chictr.org.cn/showproj.html?proj=190747, Identifier ChiCTR2300073273.

## Key points

1 The EC90 of ropivacaine for patients with obesity in labor analgesia remains unknown.
2 This study determined and compared the EC50 and EC90 of ropivacaine for patients with or without obesity in labor analgesia.
3 Obesity does not exert a significant impact on the EC50 and EC90 values of epidural ropivacaine for labor analgesia.

## Introduction

The median effective concentration (EC50) of ropivacaine for labor epidural analgesia between parturients with and without obesity have been determined and compared by prior two studies (Panni and Columb, 2006; Chen et al., 2021). These studies used the traditional up-and-down method (UDM) to determine and compare the EC50 values. It is noteworthy that EC50 may not be as clinically relevant as 90% or 95% effective concentration (EC90 or EC95), which represent concentrations providing an effective pain relief in 90% or 95% of patients, respectively. The focus of studies is therefore on the EC90 of epidural ropivacaine, which can provide pain relief for 90% of patients during uterine contractions. This measure holds greater clinical significance for labor analgesia compared to the EC50, which only offers pain relief to 50% of patients. Furthermore, UDM fails to provide insights into the shape of the dose–response curve and relative potency at different thresholds, such as EC90. In addition, the design of the UDM relies on probit for logit analyses, which are based on binary outcomes, such as “effective” and “ineffective” response, but ignored the exact pain scores that reflect parturients’ perception toward uterine contraction pain.

The dose-response relationship of epidural ropivacaine for labor analgesia has been established through multiple studies. Ngan Kee and his colleagues conducted a comparative analysis of the dose-response curves between bupivacaine and ropivacaine for labor analgesia, revealing that the 90% effective dose (ED90) of ropivacaine was determined to be 40.6 mg, which was diluted into a volume of 20 mL (Ngan Kee et al., 2010). The minimum local analgesic concentration (MLAC) model was employed by Boulier et al. using a sequential allocation method to demonstrate that the utilization of ropivacaine and sufentanil for labor analgesia resulted in an MLAC value of 0.023% (Boulier et al., 2009). The relative median potency of bupivacaine, levobupivacaine, and ropivacaine was compared by Wang et al. using probit analysis (Wang et al., 2010). It was determined that the EC95 of epidural ropivacaine was 0.214%. However, to date, limited data on the dose-response relationship of epidural ropivacaine for obese patients are available.

The present study aimed to compare the EC50 and EC90 values of epidural ropivacaine for labor analgesia between parturients with and without obesity using a traditional double-blind randomized dose-allocation method. Instead of using a binary outcome (effective or ineffective response), we quantified the response to each concentration of epidural ropivacaine based on the extent of reduction of visual analogue scale (VAS) pain scores.

## Methods

### Study design

Ethical approval for this study (IRB, K2023-0203) was provided by Hangzhou Maternity and Child Care Hospital Institutional Review Board, Hangzhou, China (Chairperson Prof Cha-Ying He) on 15 February 2023.

This is a prospective randomized double-blind dose-finding study. Before patient enrollment on 5 July 2023, we registered the clinical trial in the Chinese Clinical Trials Registry (ChiCTR2300073273). All patients provided written informed consent for study participation.

The inclusion criteria for this study were American Society of Anesthesiologists physical status Ⅱ, singleton pregnant primipara aged between 18 and 45 years, gestational age between 37 and 41 weeks, cervical dilation of 2–5 cm, visual analogue scale (VAS) pain score (0 mm = no pain, 100 mm = most severe pain imaginable) > 50 mm, and a request for labor neuraxial analgesia. The exclusion criteria were patients with uterine scarring, hypertension or preeclampsia, diabetes or gestational diabetes mellitus, obstetric complications or suspected dystocia, allergies to local anesthetics, a previous record of opioid or other sedative consumption within a timeframe of 4 h before pain relief administration, known fetal abnormalities, and any contraindication to neuraxial anesthesia.

The patients were stratified into two groups based on their body mass index (BMI): group N, patients with BMI <30 kg/m^2^, and group O, patients with BMI >30 kg/m^2^. Within each group, the patients were further randomized to receive epidural ropivacaine at concentrations of 0.0375%, 0.075%, 0.1125%, 0.15%, or 0.1875% using a computer-generated randomization schedule in Microsoft Excel (Redmond, Washington). Then, the randomization lists were concealed in opaque envelopes and opened after the enrollment of each parturient.

An anesthesiologist assistant who was aware of the patients’ allocation prepared the study solution under a sterile condition. Preservative-free saline was added to ropivacaine to make a 20-mL total volume of different concentrations of ropivacaine based on the patients’ allocation. However, the assistant was not involved in pain management and data collection.

### Procedures

Upon arrival in the labor room, noninvasive blood pressure measurement, pulse oximetry, 5-lead electrocardiography, fetal heart rate monitoring, and tocodynamometry (FM 20, Philips Medizin Systeme Boeblingen GmbH, Boeblingen Germany) were performed. Before inducing neuraxial anesthesia, a peripheral upper limb vein was established and warmed lactated Ringer’s solution (250 mL) was administered. Correct placement of the catheter at the estimated L2/3 or L3/4 vertebral interspace was confirmed through palpation with the patient in the left lateral position using the loss-of-resistance technique with saline (1–2 mL). Then, a multiorifice flexible wire-reinforced epidural catheter was inserted cephalad into the epidural space at a depth of approximately 4–5 cm. Subsequently, the catheter was gently aspirated to ensure the absence of blood or cerebrospinal fluid before securing it with a transparent tape.

After a test dose of 5 mL of the study solution, 15 mL of the study solution was additionally administered within 2 min. The patients were instructed to use the VAS pain score to report their pain level at the peak of uterine contraction during the first 30 min after the initial administration of the study solution. Simultaneously, their maternal blood pressure and heart rate as well as the fetal heart rate were monitored.

### Measurements

The primary outcome of this study is the VAS pain score 30 min after the initial dose. For patients who reported a VAS pain score >30 mm, a rescue bolus of 10-mL ropivacaine with a concentration of 0.2% was administered through the epidural catheter 30 min after the initial administration. If the VAS pain score remained >30 mm 15 min after rescue bolus administration, this indicated potential dysfunctionality of the epidural catheter; such cases were excluded from further analysis in this study. The study concluded at 30 min post-administration when satisfactory pain relief had been achieved by each patient based on their own assessment criteria for relief levels during labor contractions. Subsequent maintenance strategy regarding labor epidural analgesia was performed according to clinical practice guidelines specific to each patient’s situation as determined by the healthcare professionals included in their care team. Sensory block was assessed by evaluating changes in cold sensation using a cotton ball with alcohol. In addition, motor block was evaluated using the modified Bromage scale (Bromage, 1965) (0 = no motor block [ability to raise extended leg], 1 = ability to move feet and knees, 2 = ability to move only the feet, 3 = complete motor block). The side effects observed included maternal bradycardia (heart rate <60 bpm), hypotension (defined as systolic blood pressure (SBP) decrement ≥20% baseline or <90 mmHg; treated with intravenous phenylephrine at a dose of 50 mcg), nausea, vomiting, shivering, and pruritus. Neonatal outcomes, including 1- and 5-min Apgar scores and umbilical arterial pH, were recorded.

### Statistical analysis

The sample size of this study was determined based on a previous dose–response study (Lee et al., 2001), in which the authors identified the ED50 and ED95 values of epidural ropivacaine for labor analgesia. However, when only 15 patients were allocated to each dose, a wide range of 95% confidence intervals (CI) was observed. To achieve a narrower 95% CI, we arbitrarily increased the sample size to 20 persons per dose.

Statistical analysis was conducted using IBM SPSS Statistics for Windows version 22.0 (IBM Corp, Armonk, NY) and GraphPad Prism version 6.0 (GraphPad Software Inc., San Diego, CA). The normality or nonnormality of data distribution was evaluated using the Kolmogorov–Smirnov test. Normally distributed variables, such as age, weight, height, gestational age, cervical dilatation, and duration of the first stage of labor, were expressed as mean (standard deviation) and compared using Student’s t-test. Nonnormally distributed variables, such as sensory block level, Apgar score, *postpartum* hemorrhage, duration of the second stage of labor, and baseline VAS, were expressed as median (interquartile range) and analyzed using the Mann–Whitney *U* test to assess differences between the groups. The Jonckheere–Terpstra test was employed to evaluate the trends in sensory level and ropivacaine concentration across the subgroups. Categorical data were expressed as number (incidence) and analyzed using the chi-squared or Fisher’s exact test where appropriate. *p*-value <.05 (two-tailed) was considered to indicate statistical significance.

The EC50 and EC90 values of ropivacaine were determined via nonlinear regression analysis using GraphPad Prism 6.0. The statistical procedure was performed according to the method described in previous literature (Ngan Kee et al., 2010): the concentration values were logarithmically transformed and used as x-values, whereas response data representing pain score normalization (calculated using the following formula) were entered as y-values:

$$response\;\left(\%\right)=\frac{baseline\;VAS\;pain\;score-VAS\;pain\;score\;at\;30\;\min\;}{baseline\;VAS\;pain\;score}*100\%$$

The EC50, EC90, and 95% CI were determined via nonlinear regression analysis using GraphPad Prism 6.0. The null hypothesis was that the logEC50 or logEC90 values were similar for all datasets. Conversely, the alternative hypothesis was that the logEC50 or logEC90 values are different for each dataset. Subsequently, a comparison was made between the two groups to assess differences in the EC50 and EC90 values.

## Results

**FIGURE 1:**
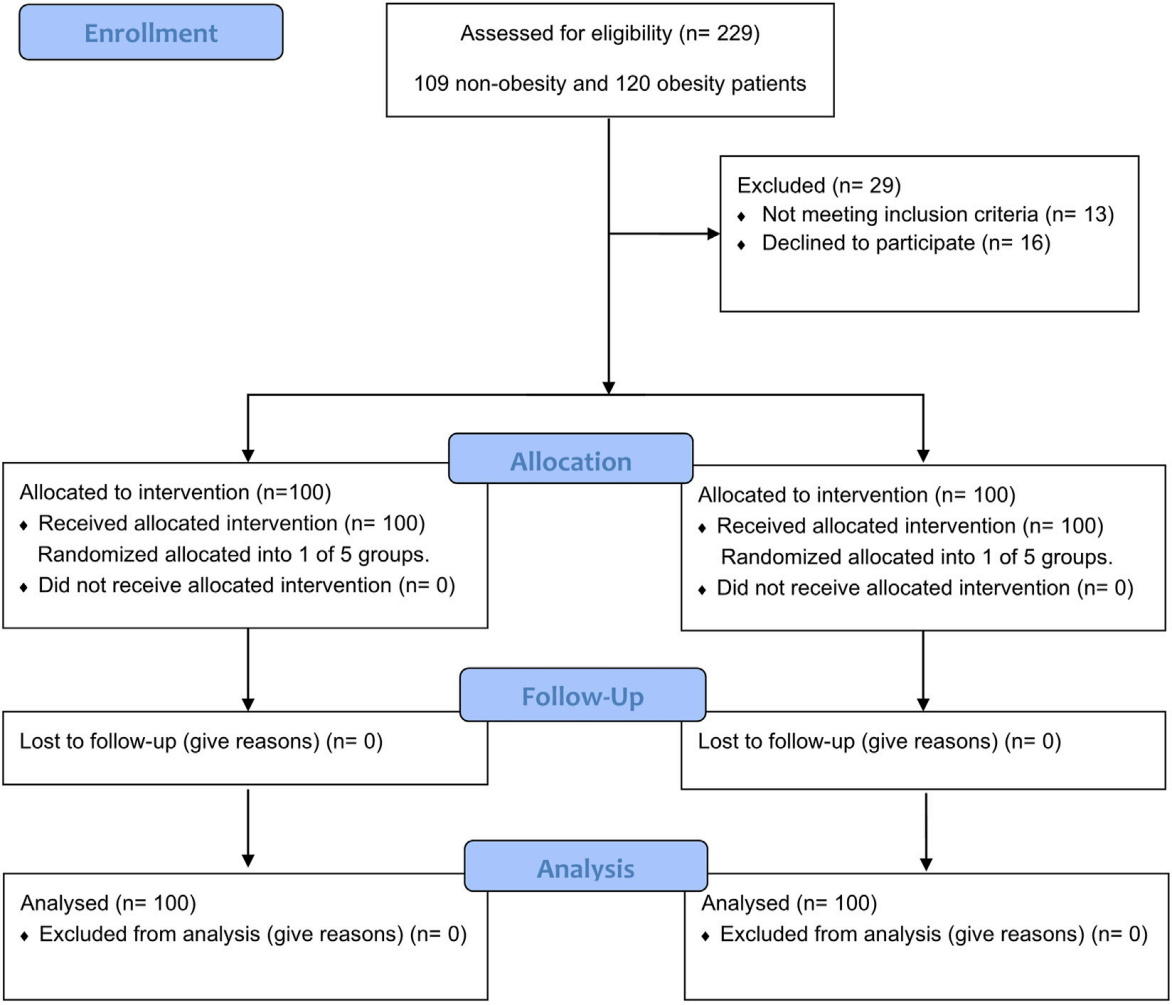
CONSORT flow diagram. CONSORT, Consolidated Standards of Reporting Trials.

This study enrolled 229 patients, including 120 and 109 patients with and without obesity, respectively. Among them, 16 declined to participate and 13 did not meet the inclusion criteria. Ultimately, 100 patients with obesity and 100 without were included in the final analysis. They were allocated into two groups and further divided into subgroups (five in each group). The Consolidated Standards of Reporting Trials (CONSORT) flow diagram is presented in Figure 1. Furthermore, the patients’ demographic and labor characteristic data are presented in Table 1. Group O had significantly higher weight and BMI values than group N (*p* < 0.001). No significant differences in other variables were observed between the two groups (all *p*-values >0.05).

**TABLE 1:** Demographic and obstetric data.

	Group O n = 100	Group N n = 100	*P*
**Age, yr**	28.24 ± 3.16	28.40 ± 3.42	0.43
**BMI**	30.91 ± 1.33	25.02 ± 2.02	<0.001
**Weight, kg**	78.07 ± 5.41	65.09 ± 5.80	<0.001
**Height, cm**	161.31 ± 4.73	160.88 ± 4.65	0.53
**Cervical dilatation, cm**	3.28 ± 0.51	3.34 ± 0.62	0.46
**Gestational age, wk**	39.07 ± 1.01	39.10 ± 0.94	0.83
**Duration of the first stage of labor, min**	611.50 ± 230.70	576.52 ± 206.68	0.293
**Duration of the second stage of labor, min**	79 (59, 116)	87 (51, 137)	0.351
**Baseline VAS (mm)**	70 (60, 80)	70 (60, 70)	0.127
**Maximum sensory level**	T8 (T6-10)	T10 (T8-11)	0.001
**Bromage Score (0/1/2/3)**	95/5/0/0	92/8/0/0	0.740
**Cesarean delivery rate**	17 (17%)	9 (9%)	0.093

Data are presented as mean ± SD, median (range) or number (%), as appropriate.

**FIGURE 2:**
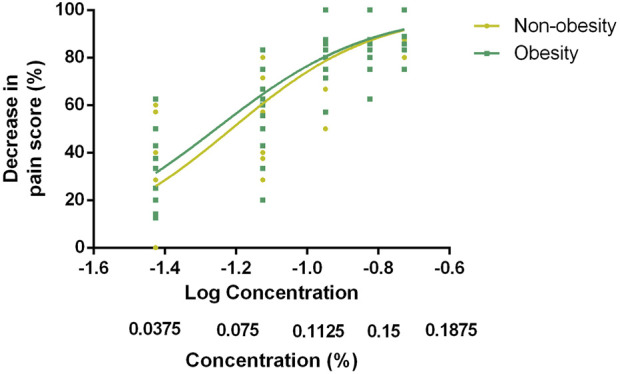
Dose–response curve of EC90 for epidural labor analgesia in parturients with and without obesity. The data was analyzed via nonlinear regression analysis, which showed EC90 values of 0.177% (95% CI, 0.152%–0.206%) in group N and 0.161% (95% CI, 0.138%–0.187%) in group O. EC90 indicates the effective concentration of ropivacaine in achieving pain relief in 90% of patients.

The EC50 and EC90 values, calculated via nonlinear regression analysis, were 0.061% (95% CI, 0.056%–0.066%) and 0.177% (95% CI, 0.152%–0.206%) in group N and 0.056% (95% CI, 0.051%–0.061%) and 0.161% (95% CI, 0.138%–0.187%) in group O, respectively. No significant difference was observed in the EC50 and EC90 values of ropivacaine between the two groups (*p*-value = 0.121 and *p*-value = 0.351, respectively). The dose–response curve for parturients with and without obesity undergoing epidural labor analgesia is presented in Figure 2. Pearson’s goodness-of-fit χ2 test revealed a good fit of the nonlinear model for both groups’ EC90 responses with *R* ^2^ values of approximately equal magnitude at around 0.75. The derived parameters for the curves are presented in Table 2.

**TABLE 2:** Calculated Parameters Derived by Fitting Variable Slope Sigmoidal Emax Dose–Response Curves to Data sets for Ropivacaine Using Nonlinear Regression.

	group N	95% CI	group O	95% CI	*P*
**Log (EC50)**	−1.213	−1.292 ∼ −1.219	−1.255	−1.249 ∼−1.177	
**EC50 (%)**	0.061	0.051–0.061	0.056	0.056–0.066	0.121
**HillSlope**	2.069	1.747–2.384	2.065	1.750–2.388	
**R** ^**2**^	0.75		0.75		
**Log (EC90)**	−0.752	−0.859∼−0.728	−0.793	−0.818∼−0.686	
**EC90 (%)**	0.177	0.138–0.187	0.161	0.152–0.206	0.351

**FIGURE 3:**
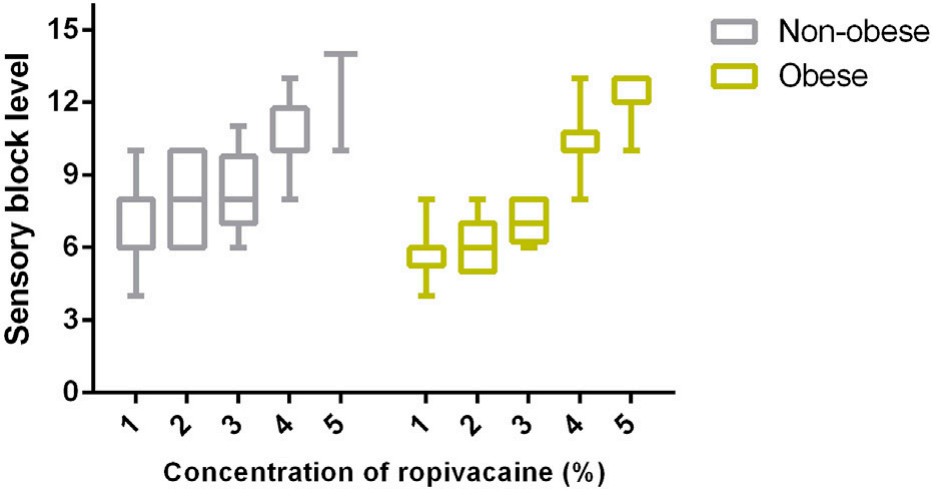
Comparison of sensory block levels between pregnant women with and without obesity using boxplots that display median, 10th, 25th, 75th, and 90th percentiles. A significant difference was observed among the subgroups within pregnant women with and without obesity (Kruskal–Wallis test; both *p* < 0.0001). Furthermore, there was a significant inverse linear trend between sensory block level and concentration within these two groups (Jonckheere–Terpstra test; both *p* < 0.001).

The baseline VAS pain score, maximum block level (variation of cold sensation, motor block level), and side effects are presented in Table 3. No differences were observed in the baseline pain scores between the two groups. The maximum block levels were T10 (T8–11) in group N and T8 (T6–10) in group O. A significant difference was observed between the two groups at this level (*p*-value = 0.001). The sensory block levels in the subgroups are presented in Figure 3, which shows a significant linear trend between sensory block level and ropivacaine concentration for both groups. The incidence of motor block was low, and no significant differences in the incidence were observed between the two groups. Furthermore, the two groups did not significantly differ in terms of side effects or cesarean delivery rates.

**TABLE 3:** Side effects during induction period and neonatal outcomes.

	Group O n = 100	Group N n = 100	*P*
**Maternal bradycardia**	0 (0)	0 (0)	--
**Hypotension**	4 (4)	5 (5)	0.756
**Nausea or vomiting**	0 (0)	0 (0)	--
**Pruritus**	0 (0)	0 (0)	--
**Shivering**	0 (0)	0 (0)	--
**1-min Apgar**	10 (9, 10)	10 (9, 10)	0.723
**5-min Apgar**	10 (10, 10)	10 (10, 10)	0.684
**Umbilical arterial pH**	7.24 (7.21–7.32)	7.25 (7.23–7.27)	0.375

Data shown as number (%), or median (quartiles) as appropriate.

There was no significant difference in neonatal outcomes, including 1- and 5-min Apgar scores.

## Discussion

In this dose–response study, we assessed and compared the EC50 and EC90 values of epidural ropivacaine for labor analgesia between patients with and without obesity. A comparable ropivacaine concentration requirement was found across these populations. In addition, we observed a two-dermatome increase in sensory block level among patients with obesity.

Obesity has recently emerged as a global health concern. Pregnant individuals with obesity are at a high risk of maternal mortality and anesthesia-related deaths, which can be attributed to the presence of underlying health conditions such as venous thromboembolism, hypertension, gestational diabetes, and obstetric complications (Lemmens, 2010). Therefore, any advancements in the prevention or treatment of obesity can greatly benefit our clinical practice. The advantage of this study lies in its comprehensive determination of the dose–response curve of epidural ropivacaine for labor analgesia in pregnant women with obesity. Unlike previous reports that determined only EC50 value as a reference for minimum effective concentration, we have also determined EC90 value, which holds more clinical relevance for routine practice. In addition, we have provided a dose–response curve (Figure 2) to provide further insights into pain relief for providers because individual responses to pain management may significantly vary and personalized labor analgesia therapy is likely to yield better outcomes.

Although the optimal dose of intrathecal local anesthetic for cesarean delivery remains controversial, research on the dose of epidural local anesthetic for labor analgesia in parturients with obesity is scarce. Panni et al. (Panni and Columb, 2006) conducted a clinical trial comparing the EC50 values of epidural ropivacaine between patients with and without obesity and determined the values to be 0.067% and 0.113%, respectively, with a significant difference observed when using the traditional UDM. Similarly, Chen et al. (Chen et al., 2021) compared the EC50 values of epidural ropivacaine for labor analgesia between patients with and without obesity using the same method and found that obesity could decrease the EC50 value. However, nonlinear regression analysis of our data revealed no significant difference in the EC50 and EC90 values between parturients with and without obesity during labor analgesia induction using epidural ropivacaine. These conflicting findings may be attributed to variations in the design methods and the statistical analyses used in different studies. The BMI of patients in Panni’s study was 39.5 kg/m2, whereas in our current study, the patients’ BMI was 30.9 kg/m2. Despite both BMI values exceeding 30 kg/m2, a significant distinction between Panni’s findings and ours emerged, suggesting that this disparity may be the primary contributing factor to the divergent conclusions drawn from these two studies. Panni et al. (Panni and Columb, 2006) employed a two-bolus induction method using 20 mL of bupivacaine (10 mL for each bolus) administered over a period of 5–10 min. In contrast, Chen et al., 2021 utilized a loading dose consisting of 5 mL of ropivacaine with dexmedetomidine at a concentration of 0.5 μg/mL (injection rate not specified) in their study. In our current investigation, we administered the study solution as a test dose (5 mL) and followed it with a loading dose (15 mL), which was delivered within 2 min. Theoretically, a relatively larger volume of local agent administered within a shorter period into the epidural space may result in more efficient spread, which could be another contributing factor leading to divergent conclusions.

Although data from this study did not indicate a variation in the effective concentration of ropivacaine for labor analgesia between parturients with and without obesity, those with obesity had a sensory block level two dermatomes higher than those without. Although this increase in block level was not associated with an elevated incidence of hypotension, T10 dermatome block is known to indicate sufficient block level for labor analgesia. Thus, we recommend reducing the volume of epidural local anesthetic rather than altering its concentration in parturients with obesity due to increased intra-abdominal pressure leading to decreased epidural space volume (Nguyen et al., 2001; Conroy et al., 2023). Consistent with our perspective, Panni and Columb also suggested that reduced epidural space volume in parturients with obesity may contribute to an elevated sensory block level after injection of local medication into the epidural space (Panni and Columb, 2006). However, the optimal volume of epidural solution for labor analgesia in pregnant women with obesity remains unclear; therefore, further studies focusing on this topic would be valuable for clinical practice in this population.

It is noteworthy that a significant linear trend was observed between sensory block level and ropivacaine concentration in this study, seemingly suggesting a potential association between sensory block level and local anesthetic density. However, we propose attributing this result to the total dose of local anesthetic administered. In our study, we used a consistent volume of 20 mL of ropivacaine with concentrations ranging from 0.0375% to 0.1875%, which correspond to total doses ranging from 7.5 to 37.5 mg. Similarly, Nakayama et al. (Nakayama et al., 2002) used lidocaine at concentrations of 1% (30 mL) and 2% (15 mL) for epidural anesthesia at the L1/2 interspace and reported similar findings regarding sensory block levels. They suggested that determination of the level of anesthesia mainly depends on the total dose of local anesthetic rather than its volume or density, which was also supported by a clinical trial involving volunteers (Liu et al., 1997).

Parturients with obesity are also at an increased risk of necessitating cesarean delivery. In a study conducted by Tabet et al., 2015, 121,092 nulliparous women were analyzed based on their prepregnancy BMI. The findings indicated that women who were overweight had an odds ratio (95% CI) of 1.50 (1.41, 1.59) for cesarean delivery whereas women with obesity had a higher odds ratio of 2.06 (1.91, 2.21). Dempsey et al., 2005 also found that pregnant women with obesity were at an increased risk of necessitating cesarean delivery. Contrary to previous studies, our findings indicate a comparable rate of cesarean delivery between women with and without obesity. This inconsistency may be attributed to several factors, including inclusion criteria favoring vaginal delivery and lower BMIs compared with other studies where BMI was considered a significant factor in intrapartum cesarean deliveries (Taylor et al., 2019; Uyl et al., 2019).

It should be noted that the outcomes of studies that utilize a binary result possess a probabilistic interpretation (Pace et al., 2007). Consequently, UDM or logit or probit regression-derived EC50 or ED50 values offer an approximation of the concentration or dose at which it is probable for 50% of the population to exhibit a response. In comparison, in investigations such as the current study in which the response is based on a graded or continuous outcome, EC50 or EC90 is an estimate of the concentration that is likely to elicit a response of magnitude that is 50% or 90% of the maximal response.

This study has several limitations. First, our observations were limited to the initiation of labor analgesia, and the EC50 and EC90 values of ropivacaine for maintaining labor epidural analgesia remain unknown. The generalizability of our results is restricted to the initial stage of labor epidural analgesia. Second, opioids are commonly used for labor analgesia in most institutions. However, we did not use opioids as epidural adjuvants owing to their potential to reduce the sparing effect of local anesthesia (Polley et al., 2000). Consequently, the concomitant use of opioids would likely decrease the EC50 or EC90 values. Third, the utilization of the initial volume of the loading dose varies across different institutions, thus it is important to acknowledge that the EC90 may vary depending on the specific volume protocol. Therefore, it should be noted that the EC90 derived from this study may not be directly applicable in other institutions.

## Conclusion

In conclusion, within the parameters of this study, our findings suggest that obesity, characterized by a mean BMI value of 30.9, does not significantly influence the EC50 and EC90 values of epidural ropivacaine for labor analgesia. Further investigations are warranted to elucidate the dose-response relationship between ropivacaine and obesity with higher BMI values.

## Data Availability

The original contributions presented in the study are included in the article/Supplementary Material, further inquiries can be directed to the corresponding author.
